# Supplementary material for: Cryo-EM structure supports a role of AQP7 as a junction protein
Source: Nat Commun. 2023 Feb 3;14:600. doi: 10.1038/s41467-023-36272-y (PMC9898259; doi:10.1038/s41467-023-36272-y)
Supplement: Supplementary file 1 — Supplementary Information [file 41467_2023_36272_MOESM1_ESM.pdf]

Supplementary Information file for

Cryo-EM structure supports a role of AQP7 as a junction protein

Peng Huang[1], Raminta Venskutonytė[1,5], Rashmi B Prasad[3], Hamidreza Ardalani[2], Sofia W de Maré[1], Xiao Fan[4], Ping Li[1], Peter Spégel[2], Nieng Yan[4], Pontus Gourdon[1], Isabella Artner[3], Karin Lindkvist-Petersson[1,5,*]

1. Department of Experimental Medical Science, Lund University, Lund, Sweden.
2. Centre for Analysis and Synthesis, Department of Chemistry, Kemicentrum, Lund University, Lund, Sweden.
3. Lund University Diabetes Centre, Clinical Research Center, Malmo, Sweden.
4. Department of Molecular Biology, Princeton University, Princeton, NJ, USA
5. LINXS - Lund Institute of Advanced Neutron and X-ray Science, Lund, Sweden.

[*]To whom correspondence may be addressed: Dr. Karin Lindkvist, Department of Experimental Medical Science, Lund University, BMC C13, 221 84 Lund, Sweden, +46 46 2228041, E-mail: karin.lindkvist@med.lu.se

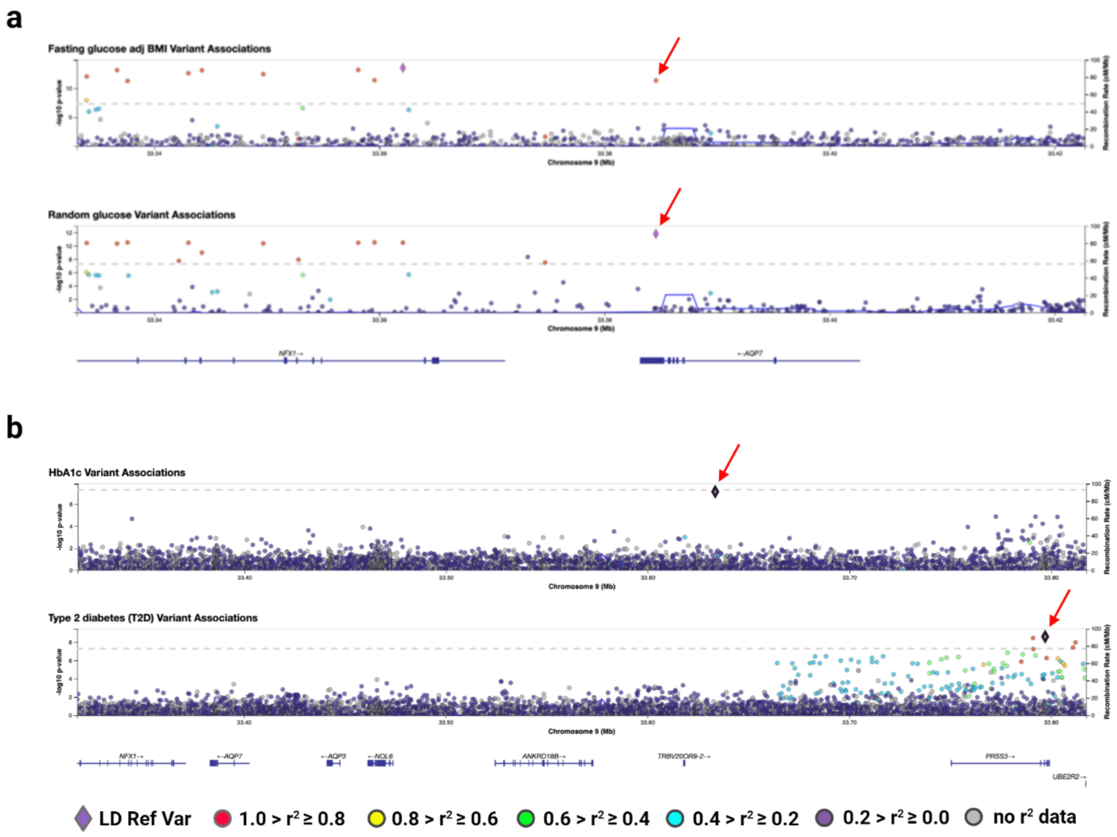

Supplementary Fig. 1 Locus zoom plot for associations of variants in the *AQP7* gene with T2D and related traits. (a) The rs2247654 variant in the 3'UTR region of the *AQP7* gene showed strong signals of association with random glucose (p=1.52×10^{-12}) and fasting glucose adjusted for BMI (p=5.08×10^{-12}) (AMPT2D portal: hugeamp.org).

(b) The SNPs rs83921 (~0.4 Mb distance of *AQP7*) and rs855532 (~0.25 Mb distance from *AQP7*) were significantly associated with T2D (p= 2.39×10^{-9})[1] and HbA1c (p= 7.42×10^{-8}) (https://www.kp4cd.org/node/120; AMPT2D portal: hugeamp.org) respectively. All SNPs mentioned are indicated by a red arrow.

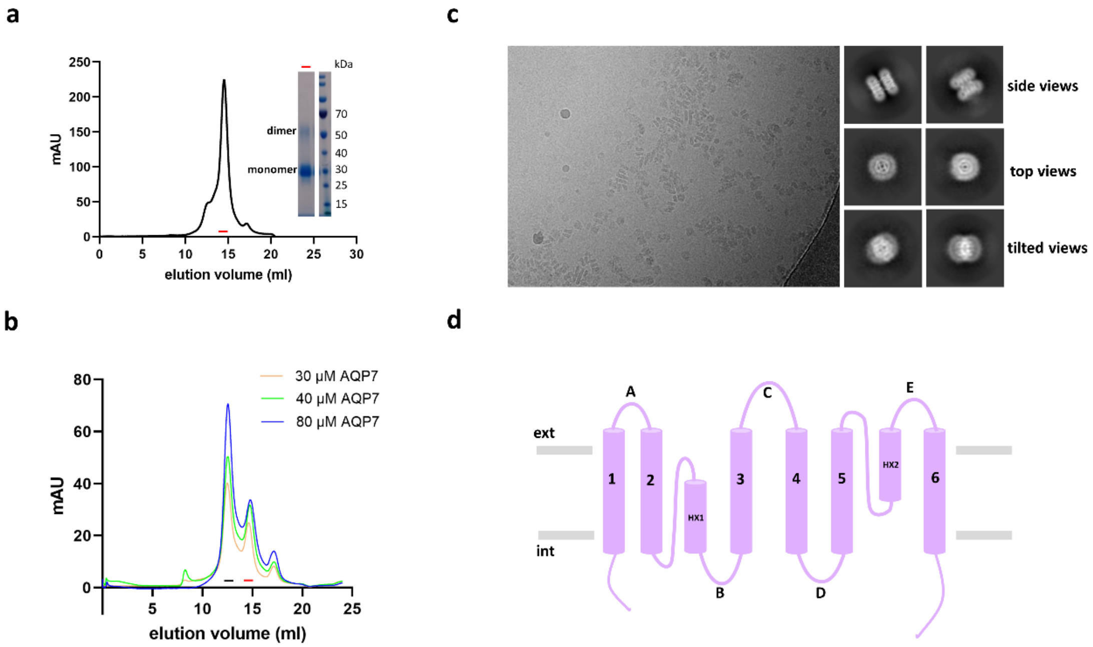

Supplementary Fig. 2 AQP7 cryo-EM sample preparation and AQP7 topological representation. (a) Size-exclusion chromatography profile of AQP7 and Coomassie-stained SDS-PAGE repeated more than 3 times independently with similar results. The fraction used for cryo-EM grid preparation was labeled by red line. (b) Size-exclusion chromatography profiles of AQP7 samples with different concentrations. The peaks in the profile corresponding to potential tetrameric and octameric oligomerizations are labeled with red and black lines in below, respectively. The chromatographic experiment was run on the same type of column (Superose 6 Increase 10/300 GL) as that used in panel a by applying AQP7 samples in different concentrations prepared in GDN detergent. (c) Representative cryo-EM image and 2D class averages in cryoSPARC[2]. This experiment has been repeated 3 times independently with similar result. (d) AQP7 topological representation. Transmembrane domains are labeled with 1-6 while two half helices by HX1 and HX2, and loops with A-E.

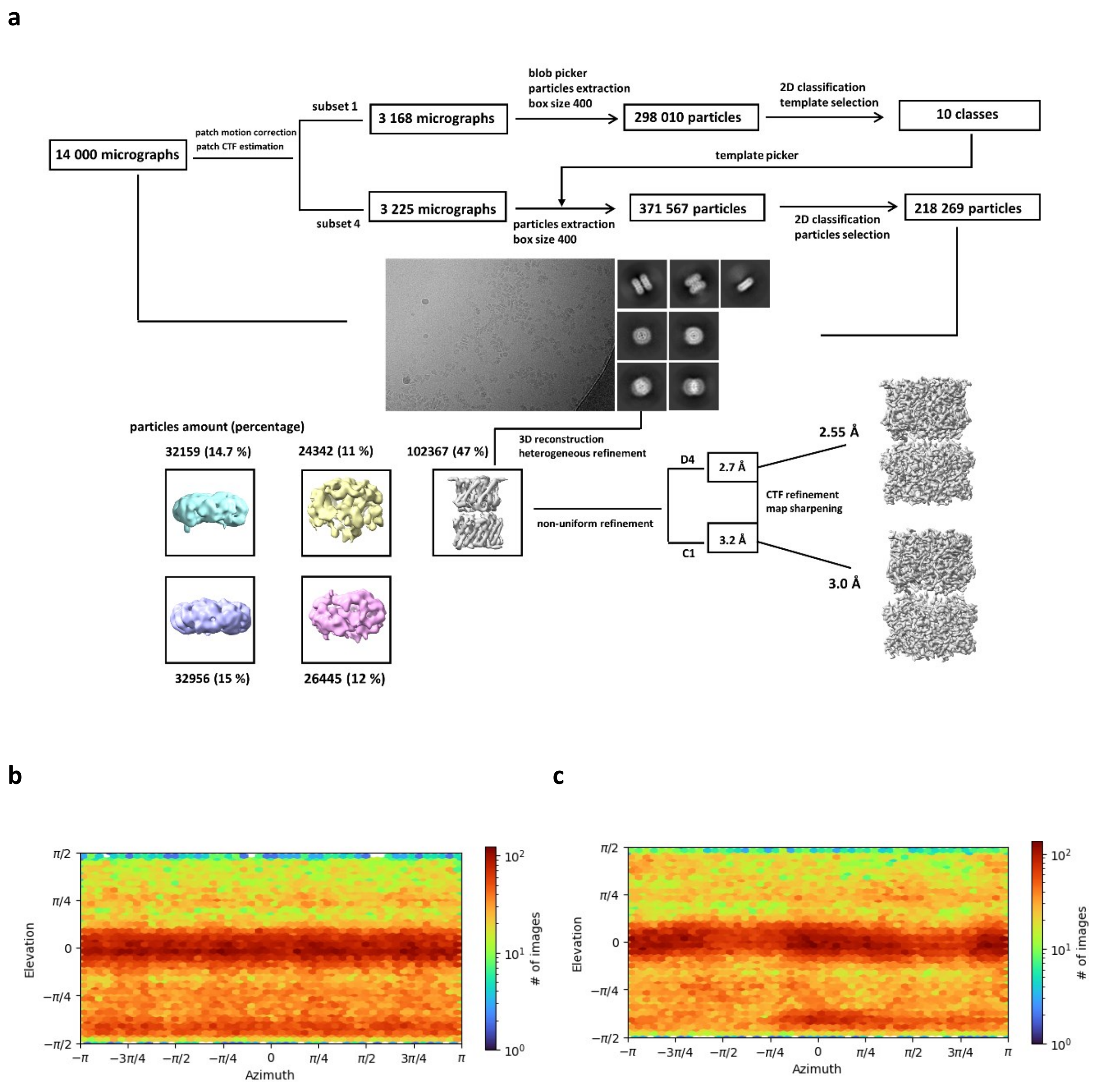

Supplementary Fig. 3 Cryo-EM data processing pipeline by cryoSPARC (a). Two AQP7 cryo-EM maps of 2.55 Å and 3.0 Å were produced with non-uniform refinement by applying D4 and C1 symmetry, respectively. Particles orientation distribution in the final 3D reconstruction for (b) D4 symmetry applied 2.55 Å map and (c) C1 applied 3.0 Å map.

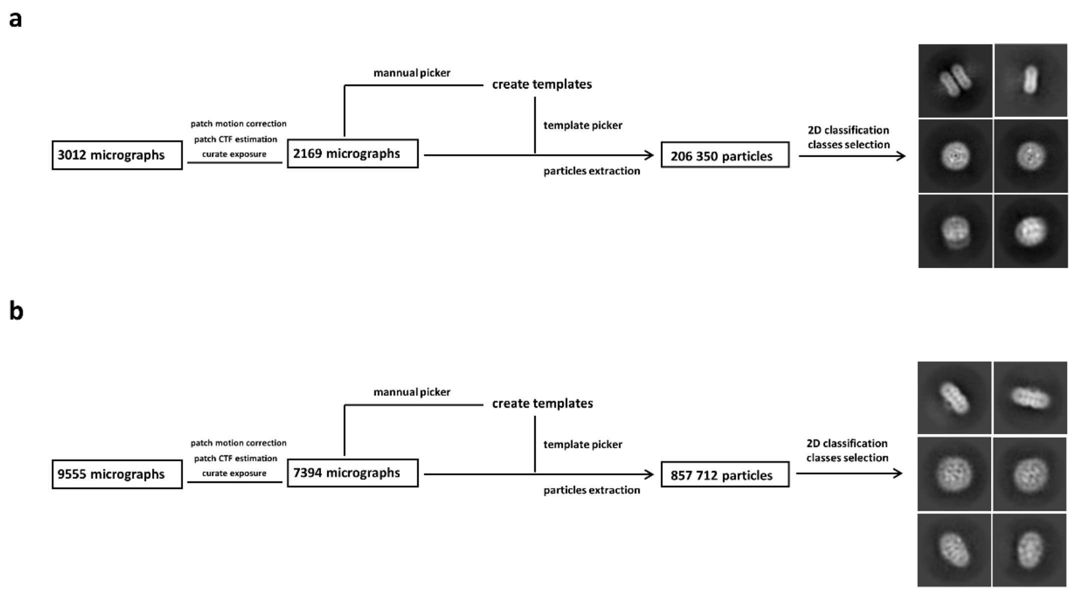

Supplementary Fig. 4 Cryo-EM data process pipeline by cryoSPARC for (a) AQP7 and (b) AQP3 collected from the C-flat Cu R1.2/1.3 300 mesh grid.

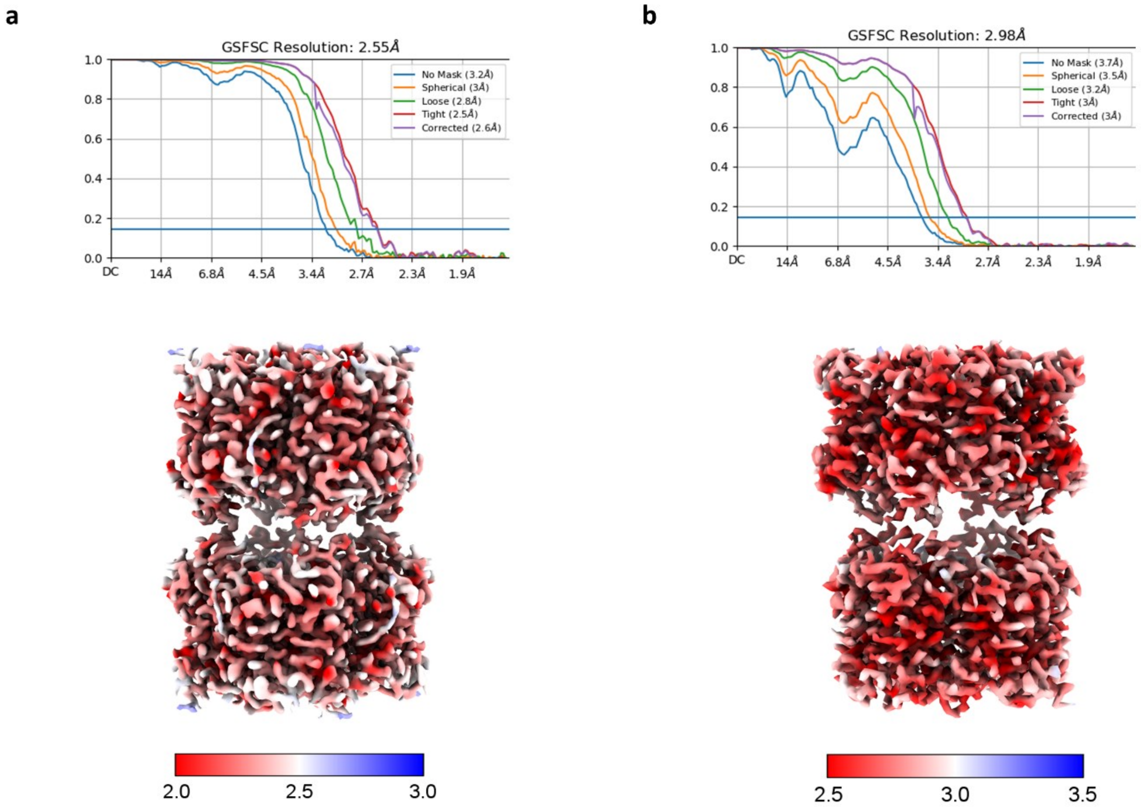

Supplementary Fig. 5 Global and local resolution analysis for (a) D4 symmetry applied 2.55 Å map and (b) C1 applied 3.0 Å map. Local resolution analysis was performed in cryoSPARC, and maps were visualized by Chimera X[3]. Indicated resolutions were estimated by gold standard Fourier shell correlation (GSFSC) at 0.143 criterion.

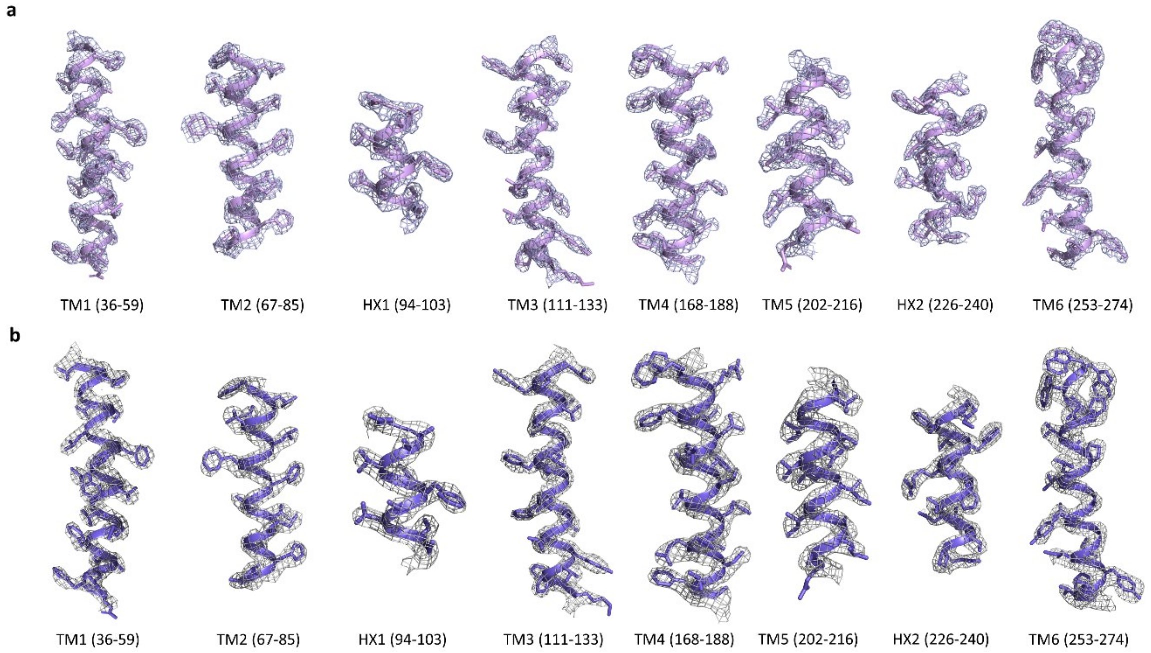

Supplementary Fig. 6 Cryo-EM map for representative fragments in AQP7. (a) Cryo-EM densities for the modelled regions from one monomer of D4 symmetry applied 2.55 Å structure and (b) from chain A of C1 symmetry applied 3.0 Å structure. Same contour level was adopted for the densities in a and b.

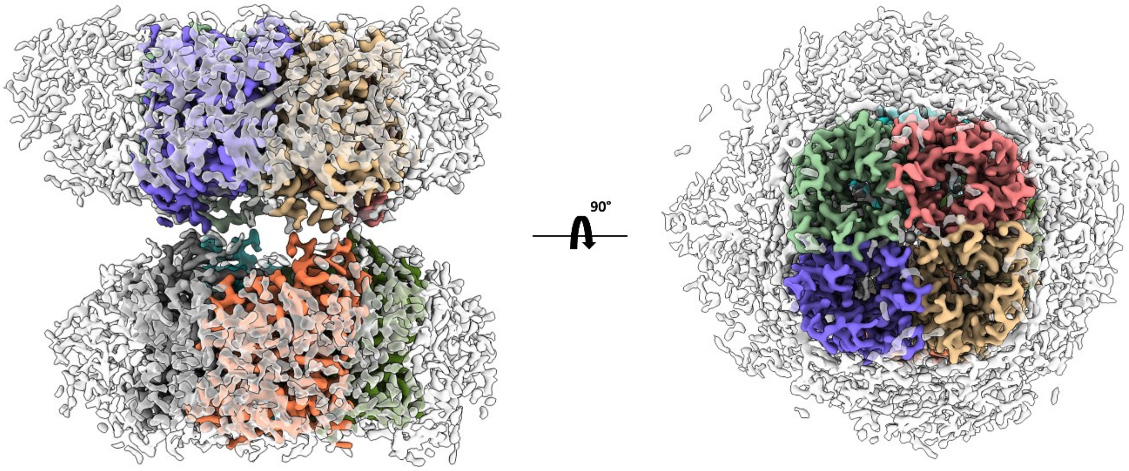

Supplementary Fig. 7 The 3.0 Å map of AQP7 dimer of tetramer encompassed in two GDN micelles. The map is colored by zone in Chimera[3].

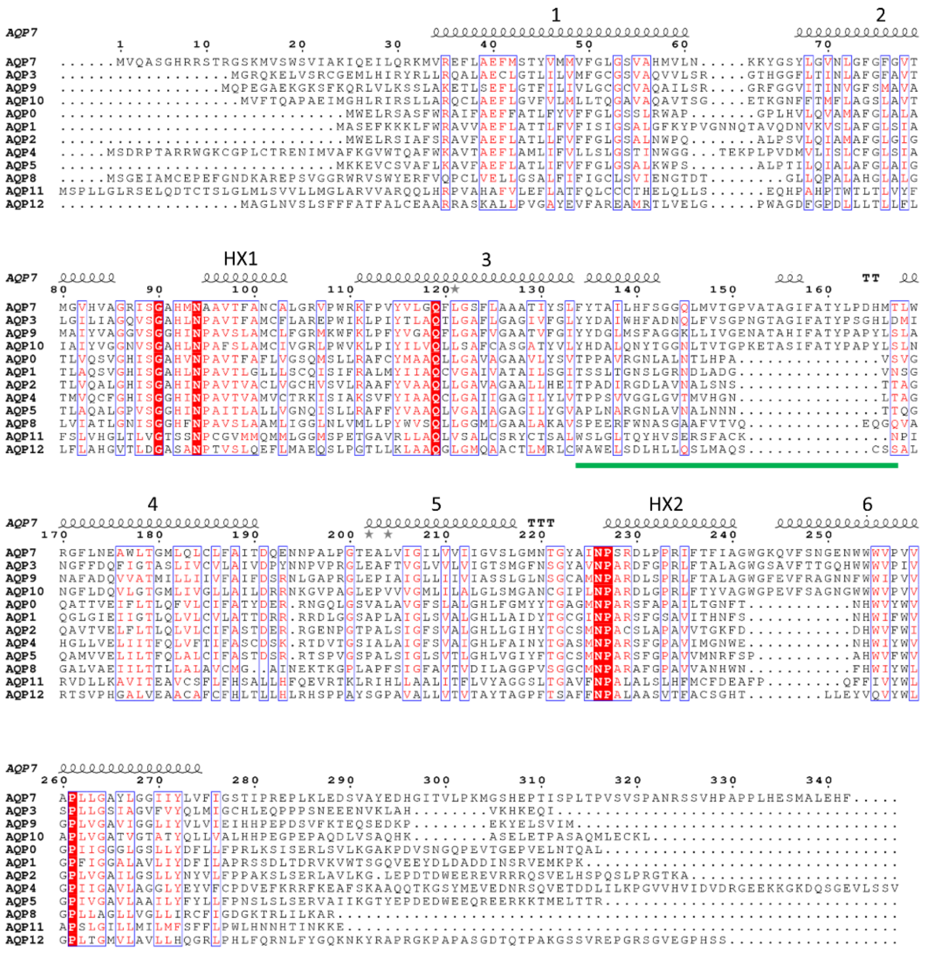

Supplementary Fig. 8 Multiple sequence alignment of human AQPs. Transmembrane segments are represented by 1-6 and two half helices by HX1 and HX2. The extracellular loop C is marked in green line. AQPs sequences were aligned by CLUSTALW and further edited by ESPript[4].

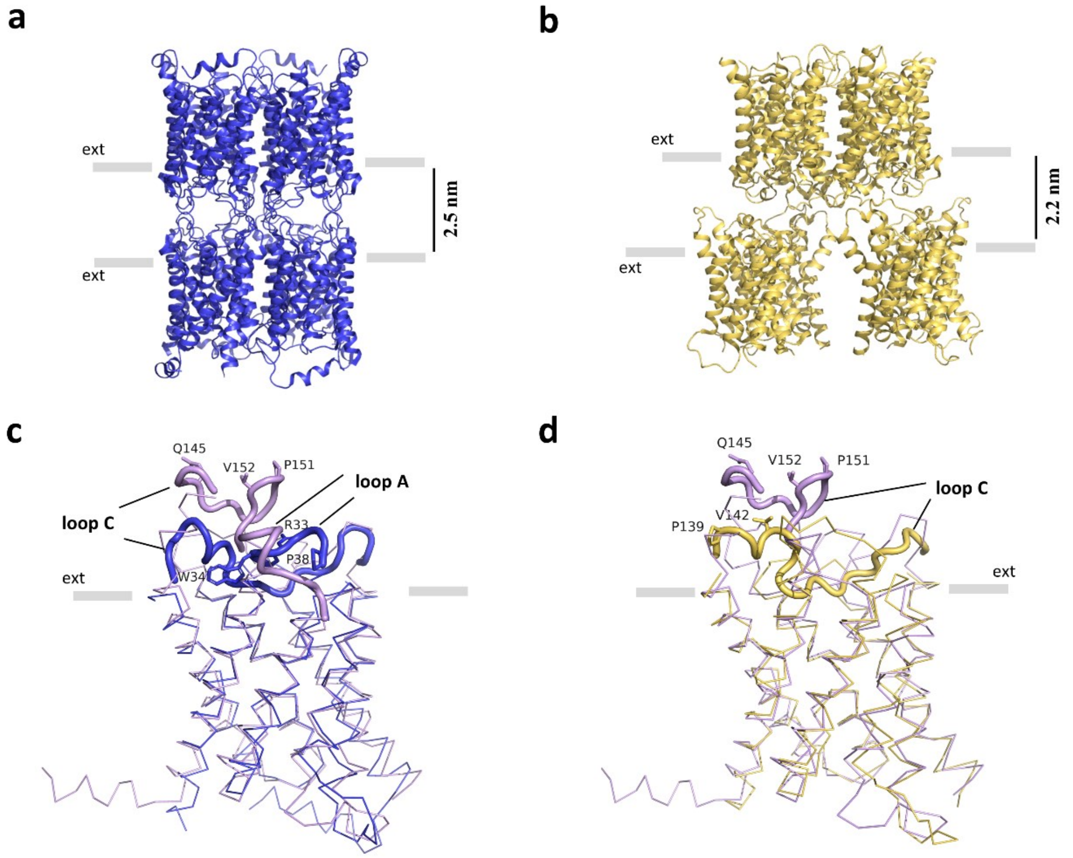

Supplementary Fig. 9 Structural comparison between AQP7, AQP0 and AQP4. The structure of (a) AQP0 (PDB ID: 2B6O) and (b) AQP4 (PDB ID: 2D57) shown as cartoon. Overlay of (c) AQP0 and (d) AQP4 to AQP7 structure, respectively. The loop C and loop A in the model are highlighted as thick tube and residues contributing to dimerization are shown as sticks.

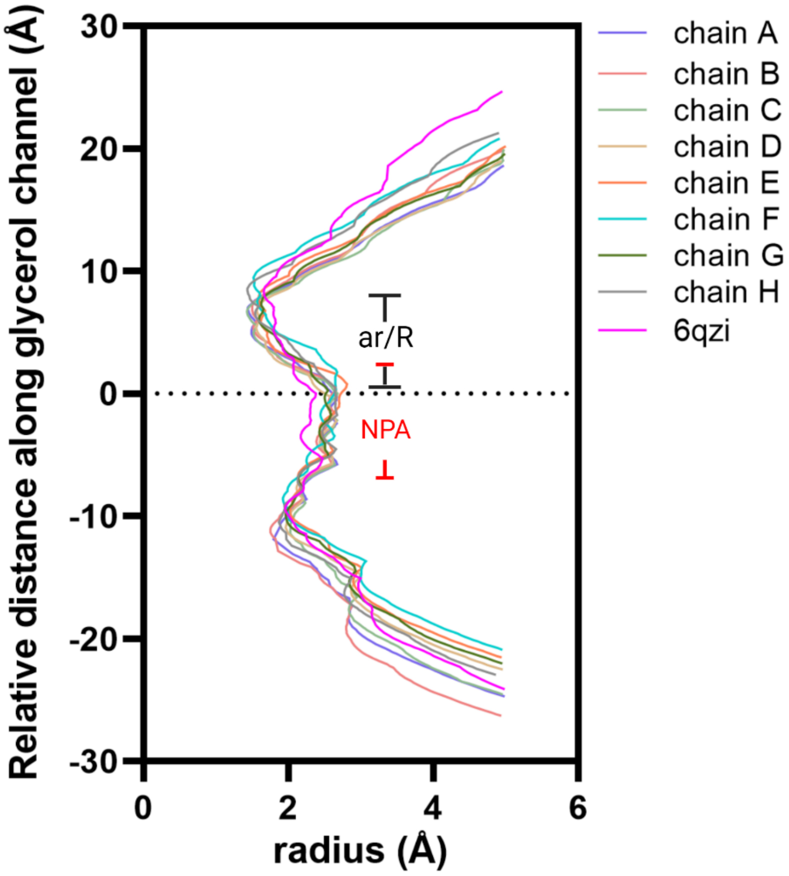

Supplementary Fig. 10 The radii along the glycerol channel (Å) were plotted for chain A-H from 3.0 Å AQP7 cryo-EM model (C1 symmetry) and the AQP7 crystal structure (PDB ID: 6qzi) calculated by the software HOLE.

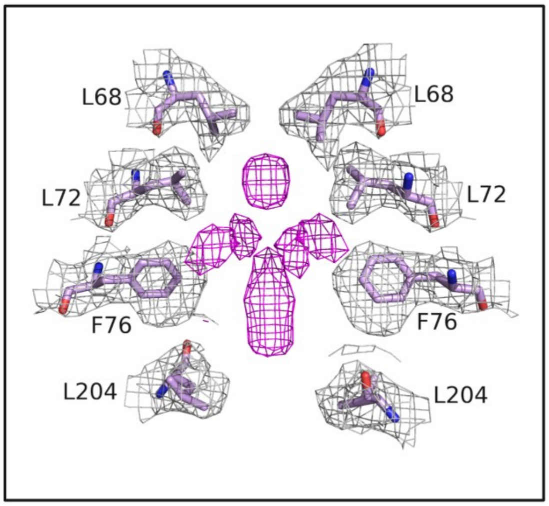

Supplementary Fig. 11 The density in the central pore in 2.55 Å D4 symmetry applied cryo-EM map. The potential ligand density and corresponding lining residues density are colored in magenta and gray, respectively, and set in same contour level.

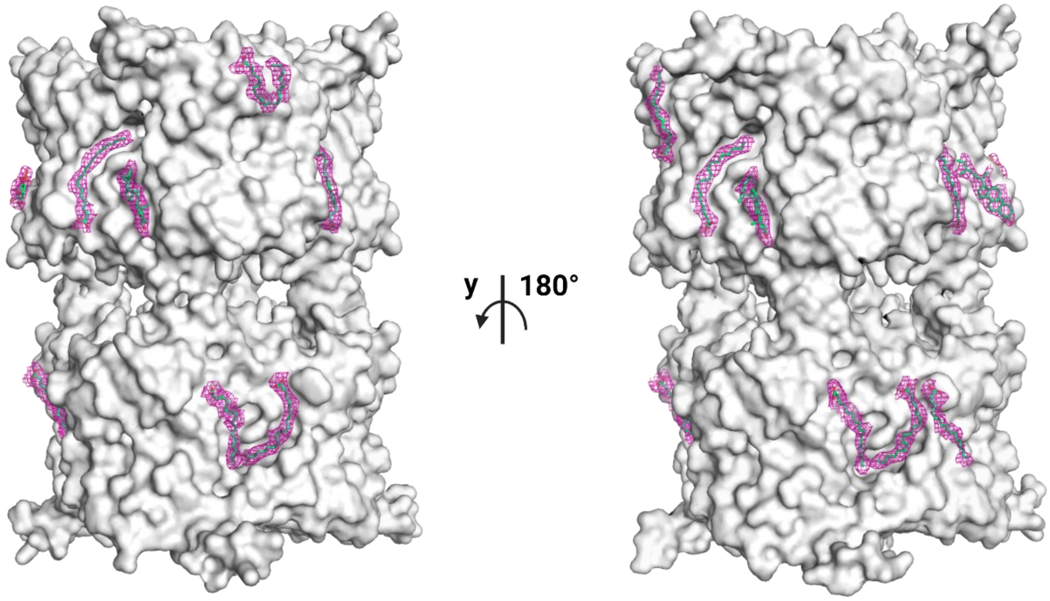

Supplementary Fig. 12 Fatty acids on the transmembrane domain surface of AQP7 dimer of tetramers. Fatty acids identified by GC/MS are fitted into the densities on the surface of AQP7 cryo-EM map. Fatty acids are shown as green sticks while the density as magenta mesh and AQP7 in grey surface.

a

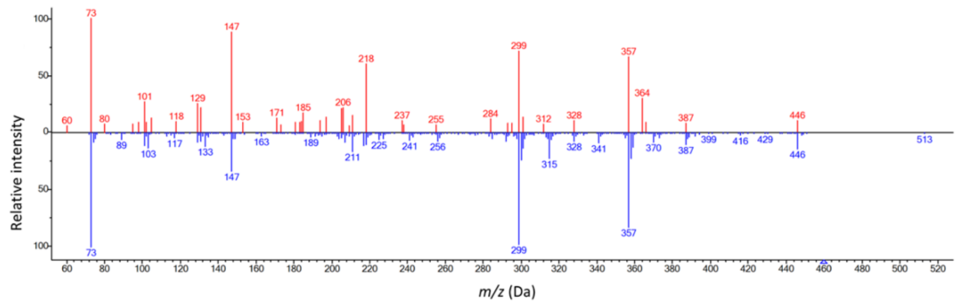

b

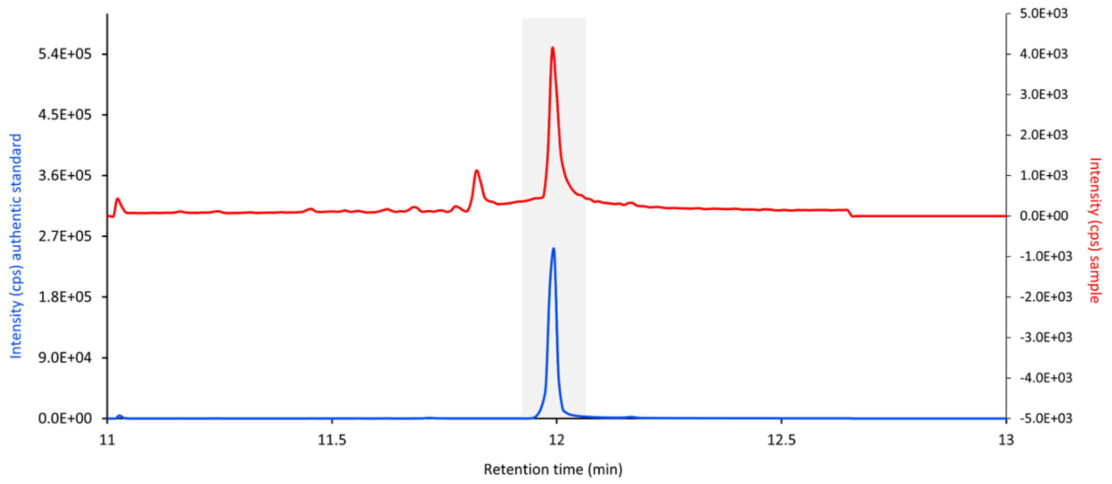

Supplementary Fig. 13 GC/MS analysis. (a) Mass spectrum for the peak recorded in full scan mode at 11.9 min in the sample (red) and mass spectrum for Gro3P from the NIST library (blue). (b) Chromatogram recorded in single ion monitoring mode at m/z 357 Da for the sample (red) and the authentic Gro3P standard (blue).

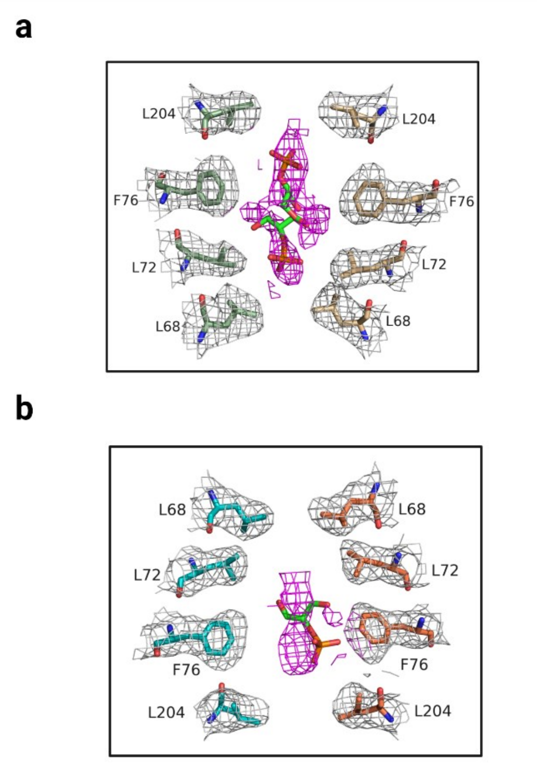

Supplementary Fig. 14 Gro2P in the density in the central pore. The central pore formed by (a) chain A-D and (b) E-H. The Gro2P molecules are shown as green sticks while the density in the central pore is shown as magenta mesh in the same contour level as lining residues density in gray.

Supplementary Table 1. Cryo-EM data collection, refinement and model statistics

	AQP7-C1-3.0 Å (PDB ID: 8AMW)	AQP7-D4-2.55 Å (PDB ID: 8AMX)
**Data collection and processing**		
Microscopy	FEI Titan Krios	
Voltage (kV)	300	
Total exposure dose (e^-/ Å^2)	50	
Frame	40	
Pixel size (Å)	0.8464	
Data process software	cryoSPARC	
Symmetry imposed	C1	D4
Map resolution (Å)	3.0	2.55
FSC threshold	0.143	0.143
**Refinement**		
Map sharpening *B* factor (Å^2)	82.2	98.6
Refinement software	PHENIX	PHENIX
Refinement tool	Real space refinement	Real space refinement
Model composition		
Non-hydrogen atoms	15792	15633
Protein residues	2032	2024
Ligands	GOL:16	0
Water	5	57
B factors (Å^2)		
Protein	57.32	48.43
Ligand	35.80	
Water	30.00	49.29
R.m.s. deviations		
Bond lengths (Å)	0.004	0.004
Bond angles (°)	0.563	0.627
Validation		
Molprobity score	1.42	1.16
Clash score	3.65	2.36
Rotamers outlier (%)	0.75	0
CaBLAM outlier (%)	1.85	2.76
Ramachandran plot		
Favored (%)	96.08	97.21
Allowed (%)	3.87	2.44
Outlier (%)	0.05	0.35

Supplementary Table 2. The alignment analysis in the representation of RMSD (root-mean-square deviation) in-between eight individual glycerol channels in C1-3.0 Å cryo-EM structure and crystal structure (PDB ID: 6qzi)

chain (glycerol channel)	A	B	C	D	E	F	G	H	6qzi
A	—	—	—	—	—	—	—	—	—
B	0.171	—	—	—	—	—	—	—	—
C	0.150	0.164	—	—	—	—	—	—	—
D	0.148	0.166	0.162	—	—	—	—	—	—
E	0.147	0.166	0.171	0.154	—	—	—	—	—
F	0.176	0.145	0.153	0.183	0.181	—	—	—	—
G	0.146	0.157	0.145	0.156	0.147	0.146	—	—	—
H	0.152	0.157	0.160	0.133	0.155	0.160	0.154	—	—
6qzi	0.572	0.573	0.572	0.579	0.569	0.579	0.561	0.566	—

(Note: — represents repeated alignments not shown in the table)

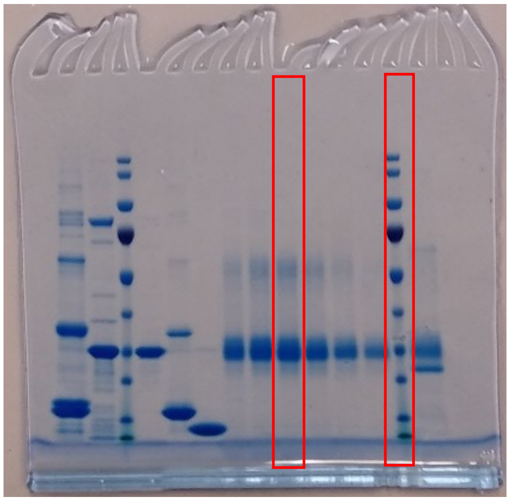

Uncropped Coomassie-stained SDS-PAGE gel shown in Supplementary Fig. 2a. The lanes presented in Supplementary Fig. 2a are labeled by red box.

Supplementary References

1 Mahajan, A. *et al.* Multi-ancestry genetic study of type 2 diabetes highlights the power of diverse populations for discovery and translation. *Nat Genet* **54**, 560-572, doi:10.1038/s41588-022-01058-3 (2022).

2 Punjani, A., Rubinstein, J. L., Fleet, D. J. & Brubaker, M. A. cryoSPARC: algorithms for rapid unsupervised cryo-EM structure determination. *Nat Methods* **14**, 290-296, doi:10.1038/nmeth.4169 (2017).

3 Pettersen, E. F. *et al.* UCSF Chimera--a visualization system for exploratory research and analysis. *J Comput Chem* **25**, 1605-1612, doi:10.1002/jcc.20084 (2004).

4 Robert, X. & Gouet, P. Deciphering key features in protein structures with the new ENDscript server. *Nucleic Acids Res* **42**, W320-324, doi:10.1093/nar/gku316 (2014).
