# Supplementary material for: Cryo-EM structure supports a role of AQP7 as a junction protein
Source: Nat Commun. 2023 Feb 3;14:600. doi: 10.1038/s41467-023-36272-y (PMC9898259; doi:10.1038/s41467-023-36272-y)
Supplement: Supplementary file 3 — Reporting Summary [file 41467_2023_36272_MOESM3_ESM.pdf]

nature portfolio

Corresponding author(s): Karin Lindkvist-Petersson

Last updated by author(s): Jan 13, 2023

Reporting Summary

Nature Portfolio wishes to improve the reproducibility of the work that we publish. This form provides structure for consistency and transparency in reporting. For further information on Nature Portfolio policies, see our Editorial Policies and the Editorial Policy Checklist.

Statistics

For all statistical analyses, confirm that the following items are present in the figure legend, table legend, main text, or Methods section.

n/a	Confirmed	
☐	☒	The exact sample size (*n*) for each experimental group/condition, given as a discrete number and unit of measurement
☐	☒	A statement on whether measurements were taken from distinct samples or whether the same sample was measured repeatedly
☐	☒	The statistical test(s) used AND whether they are one- or two-sided<br>*Only common tests should be described solely by name; describe more complex techniques in the Methods section.*
☐	☒	A description of all covariates tested
☐	☒	A description of any assumptions or corrections, such as tests of normality and adjustment for multiple comparisons
☐	☒	A full description of the statistical parameters including central tendency (e.g. means) or other basic estimates (e.g. regression coefficient) AND variation (e.g. standard deviation) or associated estimates of uncertainty (e.g. confidence intervals)
☐	☒	For null hypothesis testing, the test statistic (e.g. *F*, *t*, *r*) with confidence intervals, effect sizes, degrees of freedom and *P* value noted<br>*Give P values as exact values whenever suitable.*
☒	☐	For Bayesian analysis, information on the choice of priors and Markov chain Monte Carlo settings
☒	☐	For hierarchical and complex designs, identification of the appropriate level for tests and full reporting of outcomes
☒	☐	Estimates of effect sizes (e.g. Cohen's *d*, Pearson's *r*), indicating how they were calculated

Our web collection on statistics for biologists contains articles on many of the points above.

Software and code

Policy information about availability of computer code

Data collection	Single particle cryo-EM data were collected on Titan Krios electron microscopes with a K3 detector.
Data analysis	Cryo-EM data were processed with cryosparc (version 2.15.0, https://cryosparc.com). Model building was performed using USCF chimera 1.14, Wincoot 0.9.2 , and phenix 1.18.2. MolProbity (phenix1.18.2).The functional data was assessed using Graph Pad Prism 9 (version 9.3.0). Figures were generated using pymol (version 2.3.4) and ChimeraX (version 1.2). Alignments were performed using CLUSTALW (version 2.1), and visualized using ESPript (version 3.0).<br>The GC-MS system was controlled by MassHunter Acquisition software (version 10.0), and data deconvolution was conducted in ChemStation (version 1.0) and mass spectra similarity and the retention index for glycerol 3-phosphate were confirmed by the NIST library (version 2).

For manuscripts utilizing custom algorithms or software that are central to the research but not yet described in published literature, software must be made available to editors and reviewers. We strongly encourage code deposition in a community repository (e.g. GitHub). See the Nature Portfolio guidelines for submitting code & software for further information.

Data

Policy information about availability of data

All manuscripts must include a data availability statement. This statement should provide the following information, where applicable:

- Accession codes, unique identifiers, or web links for publicly available datasets
- A description of any restrictions on data availability
- For clinical datasets or third party data, please ensure that the statement adheres to our policy

The AQP7 model coordinates for D4 symmetry applied 2.55 Å cryo-EM structure and C1 symmetry applied 3.0 Å cryo-EM structure have been deposited in the RCSB Protein Data Bank (PDB) with the accession codes 8AMX [https://doi.org/10.2210/pdb8AMX/pdb] and 8AMW [https://doi.org/10.2210/pdb8AMW/pdb], respectively. The corresponding cryo-EM maps have been deposited in the Electron Microscopy Data Bank with the accession codes EMD-15528 [https://www.ebi.ac.uk/pdbe/entry/emdb/EMD-15528] and EMD-15527 [https://www.ebi.ac.uk/pdbe/entry/emdb/EMD-15527], respectively.
Previously published PDB codes referred in this paper: AQP7 X-ray structure with the accession code 6QZI [https://doi.org/10.2210/pdb6QZI/pdb], AQP0 X-ray structure with the accession code 2B6O [https://doi.org/10.2210/pdb2B6O/pdb] and AQP4 X-ray structure with the accession code 2D57 [https://doi.org/10.2210/pdb2D57/pdb].
Source data underlying Figures 1b-d are provided as a Source Data file along this paper. Other relevant data is available from the corresponding author with reasonable request.

Human research participants

Policy information about studies involving human research participants and Sex and Gender in Research.

Reporting on sex and gender	This has been reported in: Islet Gene View-a tool to facilitate islet research. Asplund O, Storm P, Chandra V, Hatem G, Ottosson-Laakso E, Mansour-Aly D, Krus U, Ibrahim H, Ahlqvist E, Tuomi T, Renström E, Korsgren O, Wierup N, Ibberson M, Solimena M, Marchetti P, Wollheim C, Artner I, Mulder H, Hansson O, Otonkoski T, Groop L, Prasad RB.Life Sci Alliance. 2022 Aug 10;5(12):e202201376. doi: 10.26508/lsa.202201376.
Population characteristics	The characteristics are previously published: Islet Gene View-a tool to facilitate islet research. Asplund O, Storm P, Chandra V, Hatem G, Ottosson-Laakso E, Mansour-Aly D, Krus U, Ibrahim H, Ahlqvist E, Tuomi T, Renström E, Korsgren O, Wierup N, Ibberson M, Solimena M, Marchetti P, Wollheim C, Artner I, Mulder H, Hansson O, Otonkoski T, Groop L, Prasad RB.Life Sci Alliance. 2022 Aug 10;5(12):e202201376. doi: 10.26508/lsa.202201376.
Recruitment	Cadaver
Ethics oversight	Human pancreas was obtained from the Human Tissue Laboratory, which is funded by the Excellence Of Diabetes Research in Sweden (EXODIAB) network (www.exodiab.se/home) in collaboration with The Nordic Network for Clinical Islet Transplantation Program (www.nordicislets.org). Informed consent was obtained from pancreatic donors or their relatives and all procedures were approved by the Swedish Ethical Review Authority (Permit number 2011263).

Note that full information on the approval of the study protocol must also be provided in the manuscript.

Field-specific reporting

Please select the one below that is the best fit for your research. If you are not sure, read the appropriate sections before making your selection.

☒ Life sciences ☐ Behavioural & social sciences ☐ Ecological, evolutionary & environmental sciences

For a reference copy of the document with all sections, see nature.com/documents/nr-reporting-summary-flat.pdf

Life sciences study design

All studies must disclose on these points even when the disclosure is negative.

Sample size	Complete Cryo-EM statistics are provided in the Methods section, Supplementary Table 1 and Supplementary Figures 3-7. Each structure is determined from particles from one selected grid. All samples available for human cadavers were included in the studies. The sample size chosen were based on the related literature and the number of independent experiments required for the meaningful conclusions.
Data exclusions	Complete Cryo-EM statistics are provided in the Methods section, Supplementary Table 1 and Supplementary Figures 3-7. No data was

Data exclusions	excluded.
Replication	AQP7 and AQP3 proteins production was replicated more than 3 times independently even in different conditions successfully. The formation of dimer of tetramers for AQP7 confirmed by 2D classification was reproduced and observed 3 times independently on two different type of grids.
Randomization	Extracted particles were randomly assigned to calculate gold-standard FSC. Protein sample for other assays were randomly allocated into experimental groups.<br>Tissue was collected from non-diabetic and diabetic donors. Non-diabetic donors were defined as individuals lacking the diagnosis of diabetes and with an Hba1c <42mmol/mol (6 %). Diabetic donors had been clinically diagnosed with diabetes prior to hospital admission or HbA1c above 48 mmol/mol (6.5%).
Blinding	No blinding was applied in this study.

Reporting for specific materials, systems and methods

We require information from authors about some types of materials, experimental systems and methods used in many studies. Here, indicate whether each material, system or method listed is relevant to your study. If you are not sure if a list item applies to your research, read the appropriate section before selecting a response.

Materials & experimental systems

n/a	Involved in the study
☐	☒ Antibodies
☒	☐ Eukaryotic cell lines
☒	☐ Palaeontology and archaeology
☒	☐ Animals and other organisms
☒	☐ Clinical data
☒	☐ Dual use research of concern

Methods

n/a	Involved in the study
☒	☐ ChIP-seq
☒	☐ Flow cytometry
☒	☐ MRI-based neuroimaging

Antibodies

Antibodies used	The primary antibodies used: insulin (1:1,000. Dako, Cat#506442), AQP7 (1:200. Abcam, Cat#ab32826), glucagon (1:1000. Abcam, Cat#ab10988) and somatostatin (1:200. Abcam, Cat#ab30788). Secondary antibodies used : Cy2-, Cy3-, and Cy5- conjugated α-guinea pig, α-mouse, α-rat, and α-rabbit (1:500. Jackson ImmunoResearch, Cat#s: 711-165-152, 712-176-153, 706-225-148, 715-225-150, 715-175-150).
Validation	All antibodies have been validated by respective manufacturers at least by Western blot. The secondary antibodies have been routinely used in our lab and produce reliable results with multiple primary antibodies. All antibodies have also been reported in previous studies with different species (including human and mouse) and we have confirmed expression patterns and subcellular localization.
